# Hospital Ethics Committees in accredited hospitals in Poland—availability of information

**DOI:** 10.1007/s40889-021-00134-2

**Published:** 2021-09-23

**Authors:** Patrycja Zurzycka, Grażyna Puto, Katarzyna Czyżowicz, Iwona Repka

**Affiliations:** 1 Department of Clinical Nursing, Institute of Nursing and Midwifery, Faculty of Health Sciences, Jagiellonian University Medical College, Kraków, Poland; 2 Zakład Pielęgniarstwa Klinicznego WNOZ IPiP UJCM, Ul. Kopernika 25, 31-501 Kraków, Poland; 3 Department of Internal Medicine and Environmental Nursing, Institute of Nursing and Midwifery Faculty of Health Sciences, Jagiellonian University Medical College, Kraków, Poland

**Keywords:** Hospital ethics committees, Clinical ethics, Accredited hospitals

## Abstract

The role of Hospital Ethics Committees (HECs) is to support patients and their relatives as well as medical staff in solving ethical issues that arise in relation to the implementation of medical care. In Poland there are no clearly formulated legal regulations concerning the establishment and functioning of hospital ethics committees. Hospitals applying for accreditation are obliged to present solutions defining the way of solving ethical issues in a given institution, some of them appoint HECs for this purpose. The aim of this study was to analyze information concerning the functioning of hospital ethics committees in Poland on the basis of publicly available data published on the websites of accredited hospitals. Very few accredited hospitals (56) make public information about functioning in their ethics consulting facilities through hospital ethics committees. In most cases, the information provided on the functioning of HECs is general, both in terms of the committees’ functioning, type of cases under consideration and the composition of personnel.

## Introduction

There is an increasing worldwide demand for support in solving ethical problems that arise in everyday medical care practice (Rasoal et al. 2017; Chen et al. 2014; Grönlund et al. 2016). This kind of consultation is regarded as one of the basic services that should be available in modern health care facilities (Mueller and Henriksen 2017). Hospital Ethics Committees (HECs) have been operating in health care systems for several decades (Tapper 2013). In most Central and Eastern European countries, the teams set up to carry out ethical consultations in clinical conditions were not established until 1989 (Orzechowski et al. 2020). In Poland in 2009 the Polish Bioethics Society initiated a discussion on the need to establish hospital ethics committees, initiated by the PTB statement on hospital ethics committees (Polish Bioethics Society 2009). The need to establish HECs in Polish hospitals was also recognized by the Supreme Medical Council (Supreme Medical Council 2009). Unfortunately, despite the above recommendations, the issues of HEC functioning have still not been regulated, and the functioning of ethical counseling in Polish hospitals is not the subject of research, which leads to a significant deficit of knowledge in this respect.

The aim of consultations conducted by hospital ethics committees is to identify and solve both existing and potential ethical problems related to the provided medical care, improve the quality of patient care (Wocial et al. 2016), ensure patient safety and well-being, as well as resolve conflicts between medical care personnel, patients and their relatives (Rasoal et al. 2017; Gaudine et al. 2010; Slowther et al. 2012; Pfäfflin et al. 2009). In some countries, the role of hospital ethics committees is also to evaluate and oversee clinical trials involving humans (Rasoal et al. 2017). In Poland, under the Regulation on the detailed rules for the appointment and financing as well as the mode of operation of bioethics committees 1999, such matters are handled by bioethics committees operating at district medical self-government bodies (district medical chambers), medical universities or medical research and development units (Regulation of the Minister of Health 1999).

## Regulations concerning hospital ethics committees in Poland

In Poland, there are no legal regulations obliging to establish hospital ethics committees, neither an official register of all existing HECs is maintained. Some of the medical care facilities where HECs operate have created their own (internal) regulations of functioning, based on various types of legislation in force in Poland concerning patient rights and functioning of the health care system. The lack of established and coherent solutions for solving ethical problems causes that in many institutions, ethical counselling is not available at all (there is no HEC or other form of counselling), or functions in a limited range (e.g. counselling is provided only by the agent for patients’ rights). The lack of support in solving ethical problems that arise during the provision of care has a negative impact on its quality, safety and satisfaction of patients, their relatives and personnel. Conflicts arising in the course of care between patients and their relatives and the medical personnel are usually solved on the basis of existing codes of professional ethics and laws on the exercise of a profession (e.g. physician, nurse) and legislation on patient rights. Medical personnel in problematic situations are deprived of substantive support in making decisions and solving dilemmas, usually relying on their own judgment, experience and clinical preparation. This state of affairs marginalizes institutional ethical support for problem solving, does not benefit the development of guidelines, directions, and recommendations for the future, and does not address issues related to education on how to solve emerging problems.

In the context of Polish legislation on patient’s rights, hospital ethics committees may deal primarily with issues related to the right of access to health care services, the right to information, the right to consent to proposed services (Czarkowski 2010a; The act on patient’s rights 2009). As far as the patient’s right to object to a physician’s opinion or ruling is concerned, the medical committees of the Ombudsman of Patient’s Rights are more competent than the HEC to resolve such disputes (Czarkowski 2010b). The provisions of the Act on Patient’s Rights and the Ombudsman of Patient’s Rights guarantee the right of a patient to object to physician’s opinion or ruling. A patient or his statutory representative may object to physician’s opinion or ruling (in the scope of health examination, diagnosis and prevention of diseases, treatment and rehabilitation, providing medical advice) if they affect the patient’s rights or obligations under the law (The act on patient’s rights 2009, The act on profession of physician and dentist 1997). Ethical issues related to transplantology are regulated under the Act on the collection, storage and transplantation of cells, tissues and organs. The Ethics Committee of the National Transplantation Council is the body giving its opinion on these issues. The tasks of this committee overlap with those of the HEC (The act on the collection, stotage and transplantation 2005). Some of the Polish legislation overlaps with the functions usually carried out by HEC. It would be advisable to harmonize these provisions and to establish separate legislation obliging care settings to establish HECs and setting out the principles of their functioning with reference to legislation already in force.

According to the Central Statistical Office 2018, there are 949 publicly accessible inpatient hospitals in Poland (Central Statistical Office 2018). Among them, 227 are accredited by the Center of Monitoring Quality in Health System (Center for Health Quality Monitoring 2020). The accreditation is based on voluntary application of assessment according to established and open criteria (The act on accreditation in healthcare 2009; Center for Health Quality Monitoring 2009; Regulation of the Minister of Health 2009). An institution applying for accreditation is obliged to present during the accreditation visit numerous documents, one of which is to determine the way of solving ethical issues in a given institution – ZO8 (Center for Health Care Quality Monitoring 2009). During the accreditation, the question whether mechanisms for solving ethical issues have been implemented in the hospital is verified. The set of standards for the hospital’s accreditation program includes both a review of the presented documentation concerning solving ethical issues and an interview with the management and staff of the facility. The highest scores are given to those institutions where mechanisms for resolving ethical issues have been implemented (5 points), while lower scores are given to those institutions where ethical issues have been defined but where no methods for resolving them have been developed and implemented (3 points). In order to receive accreditation it is necessary to obtain 75% of points in all analyzed areas, thus it is possible to obtain it despite the fact that an institution has not taken actions aimed at solving ethical issues (Center for Health Care Quality Monitoring 2009).

The aim of this study is to analyze information on the functioning of hospital ethics committees in Poland on the basis of publicly available data published on the websites of accredited hospitals.

## Materials and methods

The research was conducted using qualitative document analysis by means of conventional content analysis. This research technique used allowed the interpretation of the meaning of textual data content posted on hospital websites. It is a technique that is commonly used in research that aims to describe the phenomenon which has not yet been sufficiently understood (Hsieh and Shannon 2005; Graneheim and Lundman 2004). Two authors (PZ, GP) independently analyzed the content of the surveyed websites of accredited hospitals (227 facilities) in search of information regarding the functioning of hospital ethics committees. Then, relevant meaningful content was distinguished, codes were created as well as categories and subcategories were identified for further analysis and description. 56 institutions which, in the information on their websites, indicated the existence of an ethics committee (inclusion criteria) were qualified for the final study. In the next step, all fragments related to the functioning of hospital ethics committees were independently identified by two authors (PZ, GP). This made it possible to obtain several data areas: nomenclature, composition and profession of committee members, the scope of the committee's operation, the rules of conducting cases and the committee's operating procedures.

The research was conducted in the period from 01/03/2020 to 20/04/2020.

Ethics committee approval was not required because this study did not involve patients.

## Results

### Terminology

Teams were established in 56 institutions for resolving ethical problems in different ways. Most often they were called Ethics Teams (35), Ethical Teams (14), Committees for Ethics (4). The individual institutions described the teams as such: Hospital Ethics Committee, Ethics Committee of the Hospital, Hospital Ethics Team.

### Composition of the team

Among all analyzed institutions, 53.6% (30) did not provide any information on the composition of established hospital ethics committees. In 8 (14.3%) cases, no personnel were disclosed, but only information that members of the team are hospital employees. In 5 (8.9%) of these facilities, it was specified that the team includes representatives of medical and nursing circles, other representatives of medical personnel (pharmacist, psychologist, department heads) and non-medical personnel (legal counsel). In the hospitals that did not disclose the personnel of team members, no information was given on the number of team members. In 18 (32.1%) out of 56 analyzed facilities, the composition of the team including the personnel of ethical team members was given, and in 11 (19.6%) out of them the profession of a given team member (professional affiliation) was also specified. Only in case of 1 (1.8%) of the analyzed institutions, apart from the personal data and profession of the committee members, an annotation concerning education related to ethics was given – 2 members of this committee were described as bioethics. Out of 18 (32.1%) facilities that revealed the composition of committees, 9 (16.1%) of them indicated that their members are persons who also act as proxies for patient rights. Cooperation with patient representatives was declared by 10 (17.8%) of the committee if they were not part of the committee or the composition was not indicated. A small part of the institutions (14.3%) indicated that they had clergy, and (7.1%) of those institutions had given personnel of clergy, while (23.2%) of the committee included a psychologist. In the institutions that revealed the data of persons holding the commissions, the number of teams ranged from 3 to 13 people.

### Cases reported to the committee

Out of the analyzed institutions, the vast majority (91.1%) specified how people interested in contacting the ethics team can report their problems. Only 8.9% of the institutions that indicated the functioning of ethical consulting did not indicate any form of contact enabling to report the problem. The majority of facilities allow people interested in contacting the ethics team through various channels. The most preferred form of contact for service providers is electronic contact (75%). 40 (71.4%) of the facilities allow for e-mail reporting, 2 (3.6%) of them additionally allow for electronic reporting), and another 2 (3.6%) committees allow for electronic reporting only by completing and submitting the form on the facility’s website. Submitting or sending applications in writing with confidentiality rules (sealed envelope) allows 69.6% of hospitals. Some institutions (35.7%) allow for personal submission of applications and contact with committee representatives, 5 (8.9%) of the committees require prior appointment by phone. Contact by phone was possible for 25% of committees. One institution makes it possible to submit a case for further evaluation by leaving a written application in prepared for this purpose boxes located in public areas of the hospital. One institution also provides for the possibility of submitting an application to the committee orally through its member, who is obliged to prepare information from the meeting in writing; another institution provides for the possibility of submitting applications to the committee through the nurses in charge of individual departments of the hospital. Some of the facilities under analysis 28.6% specified what information should be included in the application, these were personal data of the reporting person, contact details (e-mail or correspondence address, telephone number), description of the problem situation. Nearly half of the facilities (48.2%) provided information that anonymous applications will not be considered, and 30.3% of HEC indicated that they are guided by confidentiality rules.

The majority of the institutions (78.5%) decided to include information as to who the committee should serve and who is entitled to submit applications. In 76.8 percent (43) of the cases, committees provide assistance to patients and their relatives as well as to employees (half of these committees indicated that the first place was given to help employees when listing who they were supposed to serve), and only one committee indicated that it only offers assistance to patients. As many as 76.8% of the institutions did not explicitly state who is entitled to submit applications to the committee, only 23.2% of the committees stated that they are patients and staff, in one case the patients were mentioned as entitled persons. In addition, there were single cases where volunteers, contractors, and other persons involved in the care of institution were also considered eligible. One of the institutions identified press and media reports and the results of hospital inspections and audits as a source of information on the basis of which the ethics committee can take action.

Only 9 (16%) of the facilities decided to provide information on the rules and regulations in force or the rules and procedures of the committee. In case of 7 (12.5%) institutions, the frequency of committee meetings was determined (4 quarterly, 3 semi-annually), 10 (17.8%) institutions indicated the time of application processing – it was up to 30 days (1 month). In case of 10 (17.8%) committees, the possibility of proceeding and issuing an opinion whenever such a need arises (urgent procedure) was provided for, one of the institutions indicated that a meeting of the committee must be convened within 3 days of the request being submitted.

### Scope of the committee’s activities

In terms of information on the operation of hospital ethics committees, out of 56 analyzed institutions, 26.8% only indicated that there is a unit dealing with ethical issues in the institution. The other institutions presented various detailed information on the areas that are the subject of work for the hospital ethics committees. The majority of institutions (76.8%) referred to the protection of patient rights. Nearly half of the committees (46.4%) reported that their goal is to protect patient’s rights under the Act on patient’s rights. 14.3 percent of the committees are represented by the Patient’s Rights Representatives, while 16.1 percent of the representatives are committee members. In case of 3 (5.3%) facilities, it is clarified that particular attention should be paid to protecting the rights of vulnerable patients (children, unconscious people, terminal patients, patients with mental illness). The task of 48.2% committee was to supervise the compliance of employees with the rules resulting from the codes of professional ethics and codes established by individual institutions, and 14.3% was to promote compliance with ethical principles. 75% of the committees were dedicated to providing support in resolving ethical issues for patients, their relatives and employees, 21.4% of the committees stated that their intention is to help in case of moral conflicts or ethically questionable situations. Only 12.5% of the committees were involved in answering questions about ethical issues, and determining how to resolve problematic situations. The task of 32 (57.1%) committees was to provide assistance in ethical conflict situations occurring during hospitalization between patients, their relatives and employees (as well as among the staff), while issues related to ethical issues arising from interpersonal relations were the goal of 14.3% committees. Identification and definition of ethical issues arising in the facility was the goal of only 17.8% of HECs. Educational activity in the field of ethics – promotion of knowledge on ethical conduct and conducting training in this field was the task of 15 (26.8%) committees.

According to the available information, only 28.6% of the committees stated that their task is to help in making decisions concerning eligibility for various methods of therapy and treatment. In 10.7% (6) of the cases, the committees were involved in supporting decisions regarding continuation of long-term therapy with a low chance of success – in 4 cases it was described as persistent therapy and in 2 cases as futile therapy. Problems arising from severe or fatal illness were dealt with by 8.9% (5) of the committees, and 7.1% (4) of the committees dealt with issues related to terminal care. Information from 25% of the facilities stated in general terms that the committees dealt with end-of-life issues and life-support therapy. In one of these facilities, it was clarified that these were issues related to the discontinuation of resuscitation at the patient’s request, the discontinuation of resuscitation following many earlier, short-term follow-ups, futile therapy, and terminal sedation. Ethical problems related to issues of the beginning of human life were the subject of work of only 8.9% (5) of the commission. Information from one of them specified that these problems include: pressure to carry out caesareans on non-medical indications, the unworthy handling of staff with a dead fetus, the exposure of the mother to additional suffering through inappropriate behavior and lack of tact in the statements, the problems of mothers who decide to give their baby up for adoption and leave it in the hospital ward. Other clinical practice issues considered by the committees concerned transplantology 17.8% and blood transfusions 3.6%. Among the committees surveyed, 8.9% (5) indicated that they were dealing with issues related to conducting and participating in clinical trials and medical experimentation (one of these committees dealt exclusively with clinical trials). 16% of the committees were tasked with resolving problems related to suspected employee bullying. In 5.3% (3) of the cases, the committees were tasked with taking consultative action to counteract corruption in the facility, while the committees of another 2 (3.6%) facilities consider issues related to discrimination against patients or employees. In 12.5 percent (7) of institutions, the committee may be referred to cases related to violation of professional secrecy and confidentiality.

Following an analysis of data from all 56 facilities under investigation, detailed issues were identified that were the subject of individual committees. Individual issues concerned: appointment of an expert to resolve conflicts in staff interpersonal relations, intellectual assistance in making difficult ethical decisions, assistance in finding solutions under difficult life situations, taking action beyond own capabilities or at the expense of own health (exhaustion, which poses a risk to the patient), support in making decisions based on conscientious objection, lack of respect for intimacy, inappropriate behaviour towards patients, sexual harassment. One of the committees has been involved in issuing opinions on patient discharge from the hospital where the patient is in gross violation of the order or course of the health care process and there is no concern that refusal or discontinuance of health care services may result in immediate danger to the patient’s life or the life or health of others. One committee also declared that its purpose is to conduct training in the field of legal regulations applicable to health care (concerning patients’ rights, mental health protection and transplantology).

None of the institutions under analysis presented the requirements to be met by its members, nor did it specify the rules of their appointment. None of the HECs under study presented the results of their cases or other information (statistics) summarizing their activities.

## Discussion

Since the Polish Bioethics Society initiated in 2009 a debate on the need to establish Hospital Ethics Committees (Polish Bioethics Society 2009), no legislative work has begun to normalize the functioning of such bodies in Poland. There are still no generally applicable regulations obliging hospital facilities to offer ethical consulting to patients and their relatives as well as employees. The functioning of the existing hospital ethics committees is based on general legislation concerning patients’ rights and regulations regarding the health care system functioning, as well as guidelines contained in the accreditation requirements for medical care institutions (the requirement to determine how to resolve ethical issues of ZO8) (Center for Health Care Quality Monitoring 2009).

No research is conducted in Poland on the functioning of ethical consulting in the form of hospital ethics committees. The only data known to the authors come from the research conducted in 2015 by Czarkowski et al. in which the information presented by the committee members was analyzed (survey research) (Czarkowski et al. 2015). No research has been undertaken so far to determine what information on the functioning of ethical committees is generally available.

Significant impact on the functioning of hospital ethics committees in Poland has a longstanding practice of paternalism in medical care (Orzechowski et al. 2020; Czarkowski 2010b) and the lack of legal regulations and action standards as well as HECs financing rules (Czarkowski 2010b). Also, the availability of properly educated people who could competently handle cases addressed to HECs (Czarkowski 2010a) may be important. Medical personnel is perceived as a group of professionals competent to make decisions in all dimensions of health and illness, including ethical aspects. Patients’ knowledge of the essence of ethical consulting and the possibility of obtaining help from the hospital ethics committee is still limited. Patients are insufficiently informed on the possibilities of decision support in solving ethical problems arising during medical care (Orzechowski et al. 2020; Aulisio et al. 2000; Czarkowski 2010a). This state of affairs could certainly be changed by publishing on the websites of institutions that have hospital ethics committees, comprehensive information about the essence and scope of their activities (Czarkowski 2010a). According to the statistical data, obtaining information from the Internet has become a common practice, according to the Central Statistical Office 97.3% of respondents used this source of information on a regular basis (Central Statistical Office 2019), and 47% of people searched for information related to health and illness (Eurostat 2019).

The results of own research on the composition of committees showed that over half of them (53.6%) did not indicate the personal and professional composition of its members. Among the institutions that revealed the committee’s composition, the most frequent members were medical professionals (physicians, nurses) and psychologists. Similar results were obtained by Czarkowski et al., which showed that the composition of bioethics committees, apart from physicians, included nurses 86%, psychologists 28% (Czarkowski et al. 2015), and according to the research of Prince et al. (study reports on a survey regarding recruitment, appointment, and training of members for health care ethics committees at US) – nurses were members of 98% ethics committee members (Prince et al. 2017). The research revealed that representatives of the clergy were members of only 14.3% of the commission, while according to data obtained by Czarkowski et al. representatives of the clergy were members of as many as 42% (Czarkowski et al. 2015). According to research from other countries clergy representatives were members of almost all committees at US (98%) (Prince et al. 2017), or a vast majority at UK (84%) (Slowther et al. 2012).

HECs members are expected to have the comprehensive education and skills needed to deal with cases referred to committees, having only medical education is insufficient to provide ethical consulting (Aulisio et al. 2000). According to the research of Czarkowski et al., ethical experts were members of the committee only in 2% of analyzed institutions (Czarkowski et al. 2015), similar results were obtained in own research – only in 1 (1.8%) case it was indicated that members of the committee had bioethics background. However, the research conducted by Prince et al. showed that in 85% of cases the committee members were bioethicists or philosophers. As many as 41% of surveyed teams considered education in the field of bioethics to be an important membership requirement, and 5% considered having a bioethics education to be a necessary condition for membership in the committee (Prince et al. 2017). Also, according to the results of Slowther et al. research, most ethics committees were composed of ethical experts (60%) (Slowther et al. 2012).

The declared scope of activity with regard to hospital ethics committees was analysed in own research. The committee’s task was to protect patients’ rights in accordance with applicable laws (46.4%) and to cooperate with the Patients’ Rights Commissioner (16%) to supervise compliance with ethical principles resulting from codes of professional ethics (48.2%) and to promote compliance with ethical principles (14.3%). The tasks of the Hospital Ethics Committees focused on resolving ethical problems of patients and their relatives as well as employees (75%), providing assistance in situations of moral and ethical conflict arising during hospitalization between patients, their relatives and employees (as well as among staff) (57.1%), and helping in cases of moral conflicts or ethically questionable situations (21.4%). Only 28.6% of the committees stated in general terms that their task is to assist in making decisions on eligibility for different therapies and treatments. In information provided by 25 percent of the institutions, there was a general statement that the committees deal with end-of-life issues and life-support therapy. 10.7 percent of the committees were involved in helping to make decisions about continuing therapy with a low chance of success, helping to solve problems resulting from a serious or fatal illness (8.9%), and issues related to terminal care (7.1%). Similar results in the field of functioning of hospital ethics committees were shown in Czarkowski et al. – solving problems arising in patient care (88% of analyzed institutions) and solving ethical problems related to running and implementing therapy at the end of life (futile therapy, intubation, disconnection of ventilator) 12% (Czarkowski et al. 2015). According to Slowther et al., the most common cases in which ethics committees were asked for help were decision making (88%), discontinuation of treatment (82%), patient nutrition and irrigation issues (63%), refusal of treatment expressed by the patients or their families (55%) (Slowther et al. 2012). According to research by Wasson et al., (conducted at Loyola University Medical Center, US) the committee’s activities included decision support (93.6%), issues related to the goals of care and treatment (80.8%) and issues related to the end of life (73.1%) (Wasson et al. 2016). Research conducted by Johnson et al. (conducted at trauma center in Atlanta, US) indicates that ethical committees were most frequently approached for help with end-of-life issues (47%) and joint decision making on treatment (41%) (Johnson et al. 2012).

The results of Czarkowski et al. indicate that an important role of ethics committees is to conduct medical ethics education (47%) and to resolve conflict situations in relations between employees and staff as well as patients and their relatives (88%) (Czarkowski et al. 2015). Also the study by Chen et al. (this study was conducted in three surgical intensive care units in National Taiwan University Hospital) showed that the role of hospital ethics committees was to resolve disputes between the patient’s family and healthcare representatives (37.8%) and misunderstandings between staff members (18.7%) (Chen et al. 2014). Different results were obtained in own research – educational activities involving the promotion of knowledge about ethical conduct and training in this area were the task of 26.8% of the committees. The issues related to ethical issues arising from interpersonal relations were the task of 14.3% of the committees. Moreover, the analysis of own research results revealed issues that did not appear in other studies, namely that the committee’s work also aims to solve problems related to suspected mobbing, corruption and discrimination.

## Conclusion

Out of all 227 hospitals accredited in Poland, only a small part (24.6%, 56) post on their websites information about functioning in their ethical counseling centers implemented through hospital ethics committees. In most of the analyzed cases, the information presented concerning the scope of committees’ activities is general. A small part of the institutions (46,4%) provided information about the composition and profession of ethics committee members, only one committee presented information on the ethical education of two of its members. None of the committees specified what educational, knowledge and skills requirements its members should meet.

Due to the lack of information on the activities of hospital ethics committees, as well as discrepancies in their very functioning, it seems justified for the authorized entities to take action aimed at introducing legal regulations concerning the committee’s activities as soon as possible. Current health policy in Poland regarding ethical counseling is chaotic and ineffective, lacking consistent recommendations and clear requirements for its conduct. The principles of HEC and qualifications of its members should be defined by law. Ethical counseling should be carried out obligatorily by all hospital institutions (regardless of whether they are accredited or not) and the activity of HECs should be monitored (e.g. in the form of a register of existing HECs and their obligation to submit annual reports). Also in the field of bioethics education in Poland it is necessary to introduce changes – including the broadening of bioethical issues in the teaching of medical professions (both in the form of primary and postgraduate education). The organization of widely available and free training related to bioethical issues arising in health care should be the task of both the professional self-government, the Patient Ombudsman and the institutions coordinating health care (National Health Fund, Ministry of Health).

## Limitations

Limitations of this study are based on the information provided by the analysed sources. The study covers only publicly available information provided by accredited hospitals. The comparative lists analyzed in the discussion may show limitations due to the different functions, roles and scopes of hospital ethics committees in individual countries.
